# A Pipeline with Multiplex Reverse Transcription Polymerase Chain Reaction and Microarray for Screening of Chromosomal Translocations in Leukemia

**DOI:** 10.1155/2013/135086

**Published:** 2013-10-08

**Authors:** Fei-Fei Xiong, Ben-Shang Li, Chun-Xiu Zhang, Hui Xiong, Shu-Hong Shen, Qing-Hua Zhang

**Affiliations:** ^1^School of Life Science and Technology, Tongji University, Shanghai 200092, China; ^2^National Engineering Research Center for Biochip at Shanghai, Shanghai 201203, China; ^3^Department of Hematology and Oncology, Shanghai Children's Medical Center, Shanghai Jiaotong University School of Medicine, Shanghai 200127, China; ^4^State Key Laboratory of Medical Genomics and Shanghai Institute of Hematology, Ruijin Hospital, Shanghai Jiaotong University School of Medicine, Shanghai 200025, China; ^5^Wayen Biotechnologies Inc., Shanghai 201203, China

## Abstract

Chromosome rearrangements and fusion genes present major portion of leukemogenesis and contribute to leukemic subtypes. It is practical and helpful to detect the fusion genes in clinic diagnosis of leukemia. Present application of reverse transcription polymerase chain reaction (RT-PCR) method to detect the fusion gene transcripts is effective, but time- and labor-consuming. To set up a simple and rapid system, we established a method that combined multiplex RT-PCR and microarray. We selected 15 clinically most frequently observed chromosomal rearrangements generating more than 50 fusion gene variants. Chimeric reverse primers and chimeric PCR primers containing both gene-specific and universal sequences were applied in the procedure of multiplex RT-PCR, and then the PCR products hybridized with a designed microarray. With this approach, among 200 clinic samples, 63 samples were detected to have gene rearrangements. All the detected fusion genes positive and negative were validated with RT-PCR and Sanger sequencing. Our data suggested that the RT-PCR-microarray pipeline could screen 15 partner gene pairs simultaneously at the same accuracy of the fusion gene detection with regular RT-PCR. The pipeline showed effectiveness in multiple fusion genes screening in clinic samples.

## 1. Introduction

Myeloid neoplasms and acute leukemia encompass many different clinical and pathological entities, some with unique genetic features and reflection on risk-stratification and appropriate therapy strategies. According to the World Health Organization (WHO) 2008 classification [1, 2], acute myelogeneous leukemia (AML), acute lymphoblastic leukemia (ALL), and chronic myelogenous leukemia (CML) are categorized by the presence of specific balanced chromosomal translocations: AML is associated with t(8;21)(q22;q22), inv(16)(p13q22) or t(16;16)(p13;q22), t(15;17)(q22;q12), and 11q23/*MLL* abnormalities; ALL is mainly associated with t(12;21)(p13;q22), t(9;22)(q34;q11.2), and t(1;19)(q23;p13); and CML is characterized by the Ph+ chromosome or t(9;22)(q34;q11.2). It can be said that the translocations above cover approximately 40–50% of childhood and adult AML and ALL and 90–95% of CML patients [3–6].

PCR method, especially reverse transcription-PCR (RT-PCR), nowadays has been shown to be a sensitive tool in the clinical evaluation of leukemia. As there are many distinct genetic alterations in various leukemia subtypes, it would be extremely labor intensive to evaluate specific fusions via a panel of individual monoplex assays. This can be avoided by the use of multiplex RT-PCR assays with various downstream detection methods, such as gel-based techniques and bead array [7–10]. Microarray is another useful detection assay. Two biochip-based diagnostic systems were reported: a gel-based biochip by Nasedkina et al. [11, 12] and *MLL*Fusion*Chip* and *AML*Fusion*Chip* from France [13, 14]. In these previous works, the gel-based biochip only targeted 7 chromosomal translocations, addressing 13 fusion variants in sum, while other two chips covered certain leukemia group. That was far from translocation types needed for the initial screening stage. In addition, the procedure of PCR was very complicated, with at least two parallel nested multiplex reactions. Therefore, we planned to make RT-PCR-microarray assay much easier, hoping to detect the frequently occurring and well-defined translocations in leukemia.

In our study, we described (a) the improvement of multiplex RT-PCR in combination with microarrays analysis system that facilitated the simultaneous detection of 15 chromosomal aberrations, including more than 50 mRNA splice variants with prognostic value; (b) the sensitivity level of each fusion gene in cell lines or clinical patients unique translocations; (c) the application of this method to test 200 leukemia clinical patients; and (d) the potential diagnostic value of this procedure for detection of unusual fusion genes or fusion junctions.

## 2. Material and Methods

### 2.1. Cell Lines and Patient Samples

The 15 chromosomal translocations studied and the GenBank database references for the genes involved were given in Table 1. Cell lines and some patient samples with unique translocations as positive controls were also used in the study. The leukemic cell line HL-60 served as a negative control. Cells were maintained in RPMI 1640 (Gibco, Grand Island, NY, USA) supplemented with 10% fetal bovine serum (Gibco). Meanwhile, we also constructed fusion fraction RNAs for translocations (e.g., *PLZF-RARA*, *NPM1-RARA*, *CBFB-MYH11* type D, *SIL-TAL1*, and *BCR-ABL* p230) that had neither cell lines nor positive samples. We constructed expression plasmids using pcDNA3.0 vector (Invitrogen, Carlsbad, CA, USA), then transfected the constructed expression plasmids into 293 T cells using Lipofectamine 2000 (Invitrogen), and finally collected cells in TRIzol (Invitrogen) 48 h after transfection.

200 bone marrow samples of ALL, AML, and CML patients were also used in this work for clinical screening. They were received from the Department of Hematology of Shanghai Children's Medical Center (Shanghai, China), Ruijin Hospital (Shanghai, China), and Changhai Hospital (Shanghai, China). 

### 2.2. RNA Preparation

Leukemia cell extract was prepared by the TRIzol (Invitrogen) homogenization method according to the manufacturer's recommendations. Total RNA was resuspended in RNase-free water, and its concentration and quality were determined by NanoDrop ND-1000 Spectrophotometer (NanoDrop Technologies, Wilmington, DE, USA) and by 2% agarose gel electrophoresis. Then the RNA was stored at −80°C.

### 2.3. Multiplex RT-PCR Primers

We planned to use only one multiplex RT-PCR reaction to detect more than 50 fusion gene variants of 15 chromosomal rearrangements. To reduce the number of primers used in multiple PCR, we designed chimeric forward primers and chimeric reverse primers containing both gene-specific and universal sequences. 

The gene-specific portions of the chimeric forward primers for fusion genes in leukemia were designed or modified with the primer analysis software Primer Premier 5.0 (Premier Biosoft International, Palo Alto, CA, USA), based on sequence data of fusion partner genes deposited in GenBank database or from publications [7] (Table 2). We added bacteriophage promoter T7 and SP6 to the 5′ end of the PCR amplifying forward and reverse primers, respectively, as the universal parts. Thus in the first reverse transcription step, the synthesized cDNAs could be linked to SP6 sequence at their 5′end, so that only one reverse universal primer SP6 was required in the following multiple PCR step. In addition, the universal primer, SP6, was 5′-labeled with biotin so that amplicons could be analyzed with microarrays (Figure 2). We applied *GUS* gene (beta-glucuronidase, M15182) [15] as internal positive control to avoid false-negative results cased by varying RNA quality and handling errors. Oligonucleotide primers were provided by Generay Biotechnology (Shanghai, China). 

### 2.4. Microarray Probes

**Figure 1:**
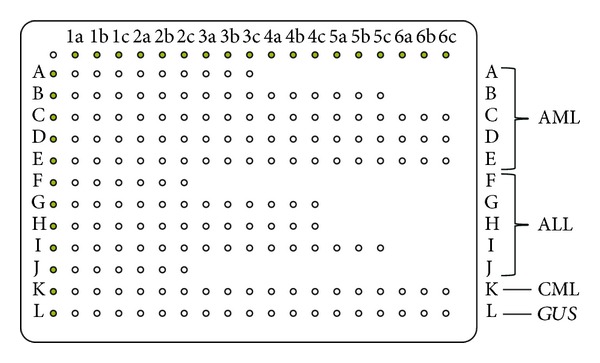
The illustration of the microarray. Left capital letters denoted the rows with specific fusion genes (A–K), and the chimeric junction probes and specific partner's probes for each fusion gene were marked by the numbers 1a, 1b, 1c–6a, 6b, 6c in triplicate. Row L represented the internal positive control *GUS* gene.

**Figure 2:**
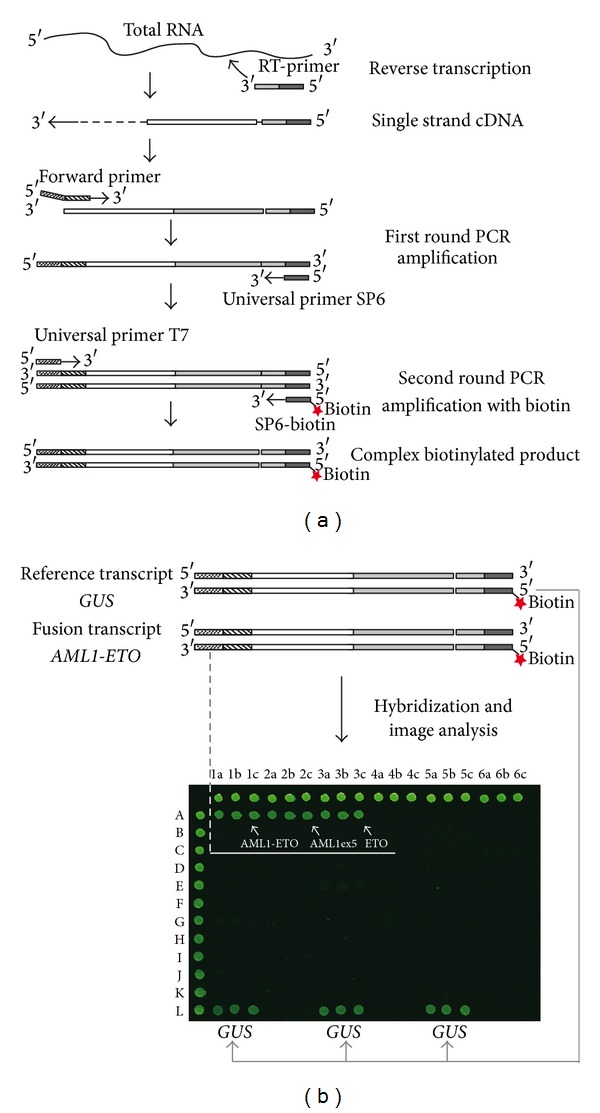
The procedure of multiplex RT-PCR-microarray assay. (a) Steps of multiplex RT-PCR. Reverse transcription was performed on total RNA with multiple chimeric reverse primers. The resulting cDNA was amplified with two-round PCR. First round was amplified with multiple chimeric forward primers on the 5′ region (upstream of known translocation points) and universal primer SP6 on the 3′ region. Second round was with universal primer T7 and biotin labeled SP6, resulting in biotinylated complex. (b) Hybridization with microarray. Take the sample with *AML1-ETO* translocation as example.

Oligonucleotide probes for translocations in leukemia were designed with the primer analysis software Primer Premier 5.0 (Premier Biosoft International), based on cDNA sequences deposited in GenBank. We designed 29 chimeric junction probes for each splice variant already known for fusion gene transcripts of interest, except the *MLL* rearrangements. In addition, 20 unique probes for each fusion genes partner were selected as positive controls in each gene fusion event, which located upstream or downstream of the described breakpoint. This microarray also included one probe for the *GUS* gene served as an internal positive control for the quality of DNA target prepared for hybridization. The sequences of probes were shown in Table 3. All probes were included in triplicate. The probes were 20–30 nucleotides (nt) long, and their specificity and sensitivity were tested by positive samples to ensure the accuracy of the microarray. The probes and biotinylated control probes were spotted onto CapitalBio optical grade aldehyde slides (CapitalBio Corporation, Beijing, China) (Figure 1). Oligonucleotide probes were synthesized in Sangon Biotech (Shanghai, China).

### 2.5. Multiplex RT-PCR

Multiplex RT-PCR was carried out in two steps. For the reverse transcription step, 2 *μ*g of total RNA was incubated at 70°C for 5 min with a mixture of the chimeric reverse primers (3 pmol of each) and then reverse-transcribed at 42°C for 1 h in a total volume of 25 *μ*L containing 200 U M-MLV reverse transcriptase (Promega, Madison, WI, USA), 25 U RNasin Ribonuclease Inhibitor (Promega), 1 mM of each dNTP, 10 mM dithiothreitol, 50 mM Tris-HCl, pH 8.3, 75 mM KCl, and 3 mM MgCl_2_. Then the cDNA reaction mixture was heated at 70°C 15 min to inactivate reverse transcriptase.

For the PCR step, a two-round PCR amplification was carried out. 1 *μ*L cDNA reaction mixture was added to 20 *μ*L of multiplex mixture containing a mixture of chimeric PCR primers (3 pmol of each), 4 pmol the universal primer SP6, 11 mM Tris-HCl, pH 8.3, 55 mM KCl, 1.5 mM MgCl_2_, 15% DMSO, 0.4 mM each of dNTPs, and 1.25 U of ExTaq HS polymerase (Takara, Dalian, LN, China). PCR cycles included 30 cycles of 95°C for 30 s, 55°C for 40 seconds, 72°C for 1 min, and finally 72°C for 7 minutes. After the first PCR, 1 uL of the first round product was added to 20 uL second-round multiplex mixtures that contained 10 mM Tris-HCl, pH 8.3, 50 mM KCl, 1.5 mM MgCl_2_, 0.2 mM of each dNTP, 0.4 pmol of forward primers T7 and 4 pmol of reverse primer SP6 (labeled with biotin), and 1 U of ExTaq HS polymerase (Takara). The second PCR consisted of 30 cycles of 95°C for 30 s, 50°C for 30 seconds, 72°C for 1 min, and finally 7 minutes of extension at 72°C. 3 *μ*L of the biotinylated-complexes was electrophoresed on an ethidium bromide stained 2% agarose gel. Negative controls without cDNA template were included for all PCR reaction mixtures.

### 2.6. Hybridization and Image Analysis

Microarray firstly was blocked in blocking buffer (2x SSC, 1 mg/mL BSA, 0.2% SDS) for 1 h at room temperature and then washed by pure water. Hybridization was performed in 25 *μ*L hybridization solution (5x SSPE, 0.1 mg/mL Salmon Sperm DNA, 0.5x Denhardt's solution, all from Invitrogen) and 20 *μ*L biotin labeled PCR products. Before hybridization, the PCR product was treated with SAP (Takara) and ExoI (Takara) to eliminate primer dimers, then denatured at 94°C, and briefly cooled on ice. Then the hybridization mixture was applied on the hybridization area covered with hybridization chamber (CapitalBio Corporation) and 12–14 h at 46°C. After incubation, the slide was washed in washing solution A (2x SSC, 1% SDS), washing solution B (1x SSC, 0.4% SDS), and washing solution C (0.6x SSC) for 5 min each, followed by incubation in Cy3-Streptavidin (SA) (Invitrogen) 1 h at room temperature. After washing, the fluorescence signal was detected on a GenePix 4000B scanner (Axon Instruments Inc., Union City, CA, USA) using 532 nm excitation. Data processing and image analysis were performed using GenePixPro6.0 software (Axon Instruments Inc.). When the averaged signal intensity from hybridization was higher than both two standard values, background value (negative samples and blank control plus 3x SD) and cutoff value (15% of biotinylated control value), we considered the probe as true signal.

## 3. Results

### 3.1. Multiplex RT-PCR and Microarray Testing

**Figure 3:**
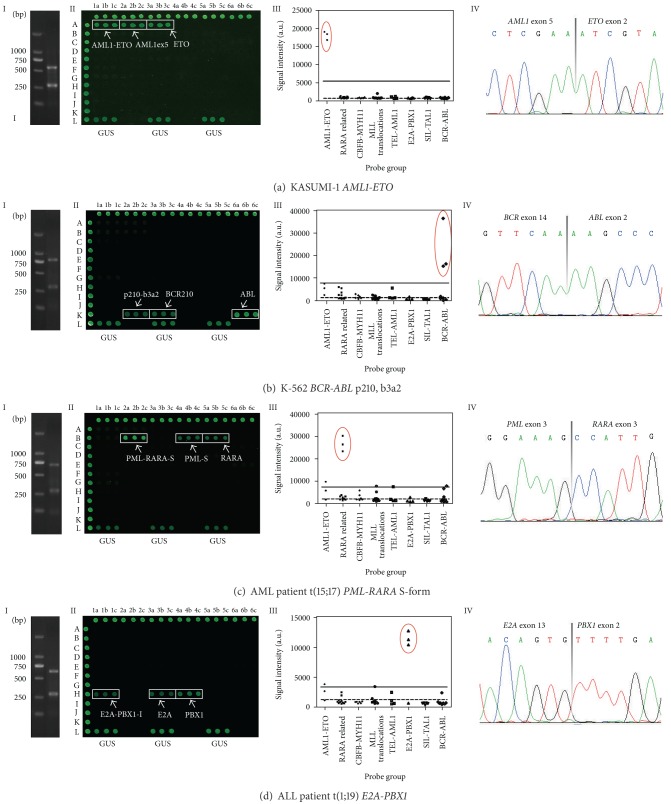
Results from the multiplex RT-PCR combined with microarray for positive controls. HL-60 and H_2_O were used as negative controls. (a) *AML1-ETO* in the cell line KASUMI-1. (b) *BCR-ABL* p210, b3a2 in the cell line K-562. (c) AML patient t(15;17) (*PML-RARA*) S-form. (d) ALL patient t(1;19) (*E2A-PBX1*). (I) Multiplex RT-PCR analysis. (II) Microarray analysis. (III) Histogram of data obtained from microarray. The solid line indicated the cutoff value (15% of biotinylated control probe signal intensity). The dashed line indicated the background value (negative samples and blank control plus 3x SD). Signals over both the cutoff and background control were recognized as true signals (in red circle). (IV) Sequence of multiplex RT-PCR products.

Positive controls, including six cell lines, eight patient positive samples of known genotypes, and five constructed fusion fractions, were used to test primer pairs and reaction conditions in the multiplex PCR reactions (Figure 3 and Supplementary Figure 1 (see Supplementary Material available online at http://dx.doi.org/10.1155/2013/135086)). The correct sequence results confirmed that we had got right specific bands by multiplex RT-PCR.

The fusion gene microarray was separated into four parts: acute lymphoblastic leukemia (ALL), acute myelogeneous leukemia (AML), chronic myelogeneous leukemia (CML), and reference control. We used *GUS *gene as an internal positive control. We divided 15 chromosomal aberrations into three groups: AML,* AML1-ETO*, *PML-RARA* and its variant rearrangements (*PLZF-RARA*, *NPM1-RARA*), *CBFB-MYH11*, and *MLL* rearrangements (*MLL-AF9*, *MLL-ENL*, *MLL-ELL*, *MLL-AF6*, and *MLL-AF10*); ALL,* MLL-AF4*, *TEL-AML1*, *E2A-PBX1*, *BCR-ABL* p190, and *SIL-TAL1*; CML, *BCR-ABL* p210 and p230. Each fusion gene included one probe for each partner and chimeric junction probes specific for each splice variant already known. The partner's probe was as positive control in each gene fusion event, which located upstream or downstream of the described breakpoint. *MLL* rearrangements only had each partner's probe. We spotted probes for each fusion gene in horizontal row, first chimeric junction probes then specific partner's probes, with continuous triplicate repeats. The biotinylated control probe was arranged in the top row and left column to position the probes and also to control fluorescence signal hybridization. After incubating in Cy3-SA, the fluorescence signal was detected on certain location.

The signal intensities of every three spotted probes were averaged. By trial and error, we confirmed that for each probe the average signal intensity from negative samples and blank control plus 3x SD was a background value of this probe. To remove the false positive signals, another standard value is established. We finally chose 15% of biotinylated control probe signal intensity as the second standard, the cutoff value, through the statistics on over 100 samples. When the signal intensity from hybridization was higher than both two standard values, the background value and cutoff value, we believed the probe as true signal. In addition, when the true signal included one chimeric junction probe and two partner's probes of the same fusion gene group, the result was considered to be positive; on the contrary, the result was considered to be negative.

Furthermore we constructed a series of plasmids for each splice variant of fusion genes studied here for optimizing all the probes. In addition, the microarray was also tested with eighteen control samples (six cell lines, eight patient positive samples, and five constructed fusion fractions) of known genotypes to check the reliability of the results (Figure 3 and Supplementary Figure 1). Figure 3 showed four examples, cell line KASUMI-1 with *AML1-ETO*, K-562 with *BCR-ABL* b3a2 splice variant, AML patient with *PML-RARA* S-form, and ALL patient with* E2A-PBX1*(I), respectively. As could be seen, the signal from the *GUS*-gene was present in all samples (row L). 

To estimate the sensitivity of our approach, a limiting dilution experiment was carried out using positive controls. RNA from all positive controls (six leukemic cells and eight patient samples with unique translocations) was serially diluted in 10^−1^ steps up to 10^−4^ with RNA from HL-60 cells carrying no translocations. Diluted samples were then used in multiplex RT-PCR, followed by microarray hybridization. For different translocations, the sensitivity assays were detected in the following dilutions: KASUMI-1 10^−3^, NB-4 10^−2^, ME-1 10^−2^, THP-1 10^−3^, REH 10^−2^, K-562 10^−2^, *AML1-ETO* 10^−3^, *PML-RARA *(S-form) 10^−2^, *MLL-AF4* 10^−2^, *MLL-ENL* 10^−2^, *MLL-ELL* 10^−2^, *MLL-AF6* 10^−3^, *MLL-AF10* 10^−3^, *E2A-PBX1* 10^−3^, and *BCR-ABL* p190 10^−3^, which indicated that the multiplex assay might detect 2–20 ng targeted RNA.

We also carried out the precision of our approach. Repeatability was tested by total RNA from one positive cell line K-562 bearing a t(9;22) *BCR-ABL* b3a2 splice variant. The RNA was divided into three parts. Each part of RNA was processed for reverse transcription and multiplex RT-PCR. Finally, the three reaction productions were hybridized onto three individual microarrays. The three hybridization results were identical, positive for the *BCR-ABL* b3a2 translocation (data not shown). Reproducibility was blindly evaluated by three different operators in three independent experiments using total RNA from cell lines (three positive, Kasumi-1, THP-1, and REH, and one negative, HL-60). The mean reproducibility of operators and experiments was 100 and 100%, respectively (Table 4). These repeatability and reproducibility results demonstrated that the multiplex RT-PCR-microarrays method had good intra- and interassay precision.

### 3.2. Patient Samples Testing

**Figure 4:**
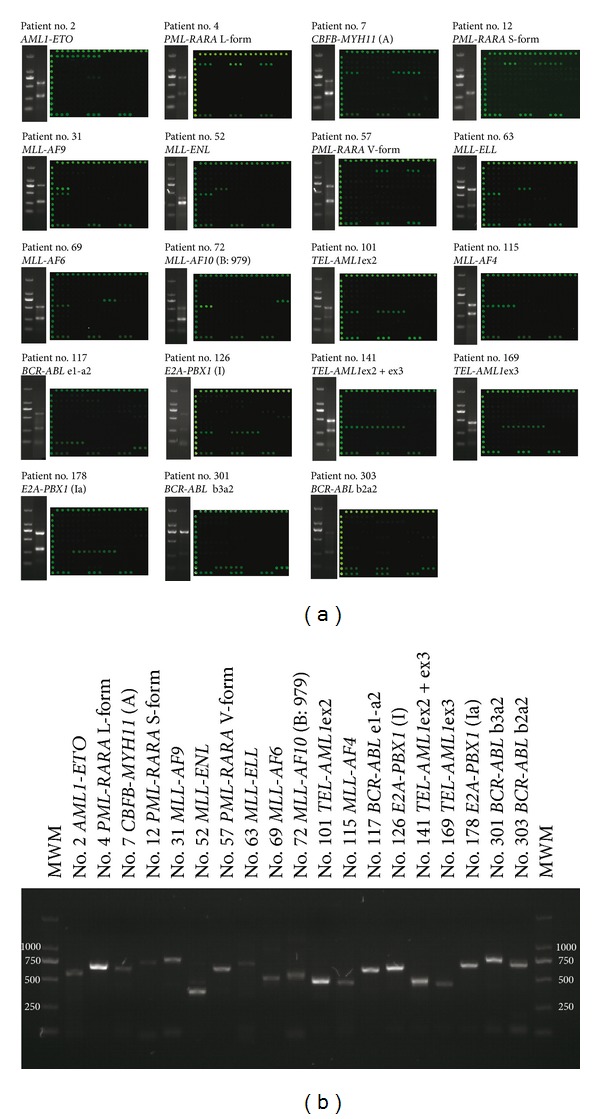
Representative results of fusion genes. (a) Multiplex RT-PCR analysis and the microarray results of 19 clinic samples (10 AML, 7 ALL, and 2 CML). HL-60 and H_2_O were used as negative controls. (b) RT-PCR validation of the microarray positive samples.

Bone marrow samples from 200 patients (74 AML, 115 ALL, and 11 CML) were analyzed blindly using the multiplex RT-PCR combined with microarrays method. Some examples were showed in Figure 4(a).

In AML patients, 37.8% chromosomal aberrations were found. Most frequent translocations were t(15;17) *PML-RARA* (13.5%), t(8;21) *AML-ETO* (10.8%), and inv(16)(p13q22) *CBFB-MYH11* (6.8%). Among ten patients with *PML-RARA*, there were five long (L)-form (*bcr1*) and four short (S)-form (*bcr3*) transcripts. It was interesting that the one remaining patient number 57 might be V-form transcript, because of the only two signals of probes PML-L and RARA besides *GUS* (Figure 4(a), patient number 57). The breakpoint of *PML* was located within exon 6 (base position 1683), belonging to *bcr2*, which was referred to as “variant” or V-form. Five *CBFB-MYH11* patients were all type A, which was consistent with previous findings that type A transcript was the major type in inv(16) positive patients.

In ALL the portion of patients carrying specific translocations was 23.5%. The most frequent translocation was cryptic translocation t(12;21) *TEL-AML1* (15.6%). In most cases, 15 of 18 patients with *TEL-AML1* were found *TEL*-*AML1*ex2 variants. Two patients had two variants *TEL*-*AML1*ex2 and *TEL*-*AML1*ex3 (weaker), owing to alternative splicing causing the skipping of *AML1* exon 2 in a minority of transcripts. The t(1;19) *E2A-PBX1 *was found in 2.6%, t(4;11) *MLL-AF4* in 3.4%, and t(9;22) *BCR-ABL* p190 in 1.7%. 

A relatively large proportion of CML patients were found t(9;22) *BCR-ABL* p210 about 72.7% including two transcripts with the b3a2 (45.4%) and b2a2 (27.2%). Perhaps, the amount of CML patients studied here was only 11, resulting in the fact that the positive portion was lower than expected.

Validation of the multiplex-microarray method was done either by cytogenetics, fluorescence *in situ* hybridization (FISH), or RT-PCR analysis for all 200 patient samples in the Department of Hematology of Shanghai Children's Medical Center, Ruijin Hospital, Changhai Hospital of Shanghai (Shanghai, China). The detailed information was listed in Supplementary Text 1 and Supplementary Table 1. The comparison of the two methods was shown in Table 5, and the concordance was about 96.5%, except four in AML and three in ALL. It was noted that these seven samples were not involved in the assay. We also performed additional individual PCR reactions for all positive samples (Figure 4(b)). The results of both methods were almost identical.

## 4. Discussion

Monoplex or multiplex RT-PCR techniques have been increasingly used to characterize chromosomal translocations found in leukemic cells, with numerous advantages over traditional cytogenetics and FISH, including shorter turn-around time, no requirement for dividing cells, and detection of cryptic translocations. A multiplex RT-PCR protocol to simultaneously detect 29 translocations had been shown to be effective for clinical screening, followed by series of identifying split-out analysis with primers specific for individual translocations [7]. It was still time- and labor- consuming. Here we facilitated the detection in a single PCR reaction (two rounds), in combination with the following microarrays to simultaneously detect a great variety of different fusion transcripts. 

We chose the 15 most frequently occurring and well-defined chromosomal rearrangements in leukemia, covering up to 40–50% of childhood and adult AML and ALL and 90–95% of CML patients. These translocations have been regarded as diagnostic and prognostic markers, reflecting risk-stratification and appropriate therapy [16–18]. In general, patients with t(12;21) *TEL-AML1* fusion and t(1;19) *E2A-PBX1* fusion in ALL as well as t(8;21) *AML1-ETO* fusion, t(15;17) *PML-RARA* fusion, and inv(16) *CBFB-MYH11* fusion in AML have the most favorable outcome [18–20], whereas those with the t(9;22) *BCR-ABL* fusion and t(4;11) *MLL-AF4 *fusion in ALL have dismal prognosis [21, 22]. *MLL* translocations are usually associated with unfavorable prognosis and poor outcome [23]. However, patients with the *MLL-AF9* translocation are clearly associated with a favorable outcome [24]. 

Compared with the previous reports [11–14, 25], the procedure established here had many advantages. First, the procedure here targeted more chromosomal translocations. We focused on about 15 chromosomal translocations, including more than 50 mRNA splice variants. However, the previous reports only had allowed the analysis of only a few rearrangements and one split variant in each fusion gene. For instance, the *AML*Fusion*Chip* [14] only included three major types of rearrangements in AML: *AML1-ETO*, *CBFB-MYH11*, and 11q23/*MLL* abnormalities (*MLL-AF9*, *MLL-ENL*, *MLL-AF6*, and *MLL-AF10*); the gel-based biochip developed by Nasedkina et al. [12] addressed 13 fusion variants in 7 leukemia translocations. Second, the range of detection was wider, covering AML, ALL, and CML. *AML*Fusion*Chip* was only limited to one leukemia group. Third, the operating steps were easier. The assay here used chimeric primers, amplifying different fusion transcripts in the same condition. In previous reports, it was necessary to perform the standard multiplex RT-PCR with multiple parallel nested PCR reactions.

A control gene was also used as an internal control to evaluate the RT-PCR reaction and hybridization process. *ABL*, *B2M*, and *GUS* were three candidate control genes for multiplex RT-PCR [15]. *ABL* is a more reliable control gene to compare diagnostic and MRD samples, yet there may be competition between *BCR-ABL *fusion transcripts and *ABL* gene when amplifying samples with t(9;22). However *GUS* gene is also similarly expressed in normal and diagnostic samples, and variation of *GUS* and fusion gene transcript expression are correlated. Based on these, we chose *GUS* as control gene in this study. 

We also tested the sensitivity of positive controls. It was the fact that the sensitivity was weak in multiplex RT-PCR compared to single PCR reactions, especially for detection of minimal residual diseases. We could observe in this report that the sensitivity of our reactions was decreased one to two orders of magnitude, due to the existence of sixteen primers in first reaction round. However, in incipient patient diagnosed with leukemia, the cell source used for RNA preparations was usually greater than 90% leukemic blasts. Moreover, the use of microarrays could improve the efficiency of discriminating splice variants and minimize the risk of contamination. Thus this multiplex PCR-microarray assay was suitable for diagnosing *de novo* leukemia.

We had scaled up in this method of analysis 200 patients with 74 AML, 115 ALL, and 11 CML. In 6 AML and 9 ALL cases, the internal control band using multiplex-microarray could not be detected. This could be ascribed to insufficient amount or quantity of RNA. Four in AML and 3 in ALL samples were beyond the detection scope of the assay. With regard to AML, t(15;17)*PML-RARA*, t(8;21)*AML-ETO* and inv(16)(p13q22)*CBFB-MYH11* were mainly identified. In ALL, t(12,21)*TEL-AML1* was predominantly observed, while t(4,11)*MLL-AF4*, t(1,19)*E2A-PBX1*, and t(9,22)*BCR-ABL* p190 were less frequently identified. Two forms of *BCR-ABL* p210 in CML were found: b3a2 and b2a2 transcripts, and the proportion of b3a2 was a little higher than that of b2a2. The majority of splice variants in *PML-RARA*, *E2A-PBX1*, and *TEL-AML1* were *PML-RARA* L-form (50%), *E2A-PBX1 *(I) (66.7%), and *TEL*-*AML1*ex2 (94.4%), respectively. In addition, we also detected a novel form of *PML-RARA*, besides L-form and S-form transcripts, classified as V-form transcripts. The statistics on the frequency of different splice variants might provide more profound and additional molecular characteristics of the majority fusion transcripts styles. This would eventually allow a more precise clinical diagnosis and optimization of therapy.

The multiplex PCR-microarray assay could also be applied to detect rare rearrangements, such as *PLZF-RARA*, *NPM1-RARA*, and *SIL-TAL1*. For *CBFB-MYH11* translocation, type A transcript accounted for more than 85% of the positive patients; two other transcripts (D and E) represented nearly 5% each, whereas all others represented unique cases [4]. We designed two reverse primers and MYH11 probes for type A and types D and E, respectively. It was indicated that there were other seven or novel transcripts though signal intensity of wild probes CBFB, MYH11-A, and MYH11-DE.

The rare chromosomal rearrangements, such as t(6;9)*DEK-CAN*, t(17;19)*E2A-HLF*, t(16;21)*TSL-ERG*, and t(3;21)*AML1-MDS1-(EVI1)*, and other *MLL* rearrangements were also worth adding the present pipeline. Although there was no independent prognostic significance, these aberrations could be very important in screening the potential chromosomal rearrangements.

## Supplementary Material

Supplementary Text 1: Methods for clinic diagnosis of 200 leukemia samples, including cytogenetic, FISH and RT-PCR analysis.
Supplementary Figure 1: Results from the multiplex RT-PCR combined with microarray for positive controls.
Supplementary Table 1: Multiplex RT-PCR - microarray and clinic diagnosis of 200 leukemia samples.

## Figures and Tables

**Table 1:** Chromosomal alterations included in the multiplex RT-PCR analysis.

Chromosome aberration	Genes involved^a^	Accession number	Positive controls		
			Cells^b^	Patients^c^	Fusion fragments
t(8;21)(q22;q22) *AML1-ETO *	*AML1* (21q22)	D43969	KASUMI-1		
	*ETO* (8q22)	D14289			
t(15;17)(q22;q21) *PML-RARA *	*PML* (15q22)	M73778	NB-4	+ (S form)	
	*RARA* (17q21)	X06538			
t(11;17)(q23;q21) *PLZF-RARA *	*PLZF *(11q23)	Z19002			+
	*RARA* (17q21)	X06538			
t(5;17)(q35;q22) *NPM1-RARA *	*NPM1* (5q35)	X16934			+ (S form)
	*RARA* (17q21)	X06538			
inv(16)(p13q22) *CBFB-MYH11 *	*CBFB* (16q22)	L20298	ME-1		+ (type D)
	*MYH11* (16p13)	D10667			
t(12;21)(p13;q22) *TEL-AML1 *	*TEL *(12p13)	U11732	REH		
	*AML1* (21q22)	D43969			
t(1;19)(q23;p13) *E2A-PBX1 *	*E2A* (19p13)	M31222		+	
	*PBX1* (1q23)	M86546			
t(9;22)(q34;q11) *BCR-ABL *	*BCR* (22q11)	X02596	K-562 (p210)	+ (p190)	+ (p230)
	*ABL* (9q34)	X16416			
del(1)(p32;p32) *SIL-TAL1 *	*SIL* (1p34)	M74558			+ (type I)
	*TAL1* (1p34)	S53245			
t(4;11)(q21;q23) * MLL-AF4 *	*MLL* (11q23)	L04284		+	
	*AF4* (4q21)	L13773			
t(9;11)(p22;q23) *MLL-AF9 *	*MLL* (11q23)	L04284	THP-1		
	*AF9 *(9p22)	L13744			
t(11;19)(q23;p13.3) *MLL-ENL *	*MLL* (11q23)	L04284		+	
	*ENL* (19p13.3)	D14539			
t(11;19)(q23;p13.1) *MLL-ELL *	*MLL* (11q23)	L04284		+	
	*ELL* (19p13.1)	U16282			
t(6;11)(q27;q23) *MLL-AF6 *	*MLL* (11q23)	L04284		+	
	*AF6 *(6q27)	U02478			
t(10;11)(p12;q23) *MLL-AF10 *	*MLL* (11q23)	L04284		+	
	*AF10* (10p12)	U13948			

^a^Chromosomes on which genes are located are in brackets.

^b^Cells were kindly provided by Ruijin Hospital (Shanghai, China).

^c^Patients RNAs were kindly provided by Shanghai Children's Medical Center (Shanghai, China).

**Table 2:** Chimeric primers used in the multiplex RT-PCR.

Primer name	Primer composition (size)^a^	Sequence (5′ to 3′)
ETO_R	D14289_SP6_614 (18)	ATTTAGGTGACACTATAGA GAACTCTTTCTCCTATCT
RARA_R	X06538_SP6_696 (16)	ATTTAGGTGACACTATAGA CGGTCGTTTCTCACAG
MYH11-A_R	D10667_SP6_2271 (18)	ATTTAGGTGACACTATAGA TTGCGTAGCTGCTTGATG
MYH11-DE_R	D10667_SP6_1374 (15)	ATTTAGGTGACACTATAGA GCAGGCTGTTCCGCT
AML1_R	D43969_SP6_731 (17)	ATTTAGGTGACACTATAGA CACGGAGCAGAGGAAGT
PBX1_R	M86546_SP6_657 (19)	ATTTAGGTGACACTATAGA TCGCAGGAGATTCATCACG
TAL1_R	S53245_SP6_257 (15)	ATTTAGGTGACACTATAGA CGTCCCTCTAGCTGG
ABL_R	X16416_SP6_576 (17)	ATTTAGGTGACACTATAGA AGCTGCCATTGATCCCG
AF9_R	L13744_SP6_1910 (20)	ATTTAGGTGACACTATAGA TTCTTGATGCATCCAGTTGT
ENL_R	D14539_SP6_301 (20)	ATTTAGGTGACACTATAGA GACCACCTTCTCCACGAAGT
ELL_R	U16282_SP6_461 (17)	ATTTAGGTGACACTATAGA GTAGCGGCCTCCAGCCT
AF6_R	U02478_SP6_360 (18)	ATTTAGGTGACACTATAGA AATCTGCCTTCCCGATCA
AF10-A_R	U13948_SP6_2384 (19)	ATTTAGGTGACACTATAGA CACTGCCTCTCCAAAAGCT
AF10-B_R	U13948_SP6_1146 (18)	ATTTAGGTGACACTATAGA TGACCTGAGCTGTGAGCT
AF4_R	L13773_SP6_1674 (18)	ATTTAGGTGACACTATAGA TCGAGCATGGATGACGTT
GUS_R	M15182_SP6_2057 (18)	ATTTAGGTGACACTATAGA TGCCGTGAACAGTCCAGG
AML1_F	D43969_T7_903 (22)	TAATACGACTCACTATAGGGA CCAGGTTGCAAGATTTAATGAC
PML-L_F	M73778_T7_1438 (19)	TAATACGACTCACTATAGGGA CAGTGTACGCCTTCTCCATCA
PML-S_F	M73778_T7_927 (25)	TAATACGACTCACTATAGGGA GTGCGCCAGGTGGTAGCTC
PLZF_F	Z19002_T7_1092(21)	TAATACGACTCACTATAGGGA CCACAAGGCTGACGCTGTATT
NPM1_F	X16934_T7_160 (25)	TAATACGACTCACTATAGGGA ACGAAGGCAGTCCAATTAAAGTAAC
CBFB_F	L20298_T7_267 (22)	TAATACGACTCACTATAGGGA TTTGAAGGCTCCCATGATTCTG
TEL_F	U11732_T7_871 (23)	TAATACGACTCACTATAGGGA CACTCCGTGGATTTCAAACAGTC
E2A_F	M31222_T7_1243 (22)	TAATACGACTCACTATAGGGA AAGATAGAAGACCACCTGGACG
SIL_F	M74558_T7_24 (19)	TAATACGACTCACTATAGGGA CGACCCCAACGTCCCAGAG
BCR-190_F	X02596_T7_1590 (20)	TAATACGACTCACTATAGGGA CGCTCTCCCTCGCAGAACT
BCR-210_F	X02596_T7_2952 (24)	TAATACGACTCACTATAGGGA GAGTCACTGCTGCTGCTTATGTC
BCR-230_F	X02596_T7_3682 (19)	TAATACGACTCACTATAGGGA CCAAGGTGCCCTACATCGT
MLL_F	L04284_T7_3916 (20)	TAATACGACTCACTATAGGGA CCGCCTCAGCCACCTACTAC
GUS_F	M15182_T7_1786 (20)	TAATACGACTCACTATAGGGA GGAATTTTGCCGATTTCATG
T7		TAATACGACTCACTATAGGGA
SP6		ATTTAGGTGACACTATAGA
SP6-biotin		biotin-ATTTAGGTGACACTATAGA

_R: reverse primer, _F: forward primer.

^a^Primer composition was given with gene accession number plus universal gene (T7 or SP6), the starting position on the gene, and total length in brackets.

**Table 3:** Sequence of oligonucleotide probes used on the microarray.

Chromosome aberration	Gene name	Fusion variants	Probe name	Sequence (5′ to 3′)	Position on microarray
t(8;21)(q22;q22)	*AML1-ETO *	*AML1-ETO *	AML1-ETO	CCCGAGAACCTCGAAATCGTACTGAGAAG	A-1abc
			AML1ex5	CTCAGGTTTGTCGGTCGAAGTGGAAGAGG	A-2abc
			ETO	GCCAGACTCACCTGTGGATGTGAAGACGCAA	A-3abc

t(15;17)(q22;q21)	*PML-RARA *	L-form	PML-RARA-L	CCGGGGAGGCAGCCATTGAGACCCAGAG	B-1abc
		S-form	PML-RARA-S	CACCCAGGGGAAAGCCATTGAGACCCAGAG	B-2abc
			PML-L	CAGAAGAGGAAGTGCAGCCAGACCCAGTGCC	B-3abc
			PML-S	AGAGGATGAAGTGCTACGCCTCGGACCAG	B-4abc
			RARA	CCCTCTACCCCGCATCTACAAGCCTTGCTT	B-5abc

t(11;17)(q23;q21)	*PLZF-RARA *	*PLZF-RARA *	PLZF-RARA	CTTACTGGCTCATTCAGCCATTGAGACCCAGA	C-1abc
			PLZF	GGGATGAAGACGTACGGGTGCGAGCTCTG	C-2abc

t(5;17)(q35;q22)	*NPM1-RARA *	L-form	NPM1-RARA-L-1	AGGAGGAGGATGTGAACAGGGTTTTATTTATGAA	C-3abc
		S-form	NPM1-RARA-L-2	GTTGGAAATTGGCAGCCATTGAGACCCAGA	C-4abc
			NPM1-RARA-S	GTGGACAGCACTTAGTAGCCATTGAGACCCAGA	C-5abc
			NPM1	ACACCACCAGTGGTCTTAAGGTTGAAGTGTGG	C-6abc

inv(16)(p13q22)	*CBFB-MYH11 *	A	CBFB-MYH11-A	CGGGAGGAAATGGAGGTCCATGAGCTGGAGA	D-1abc
		D	CBFB-MYH11-D	CGGGAGGAAATGGAGAATGAAGTTGAGAGCG	D-2abc
		E	CBFB-MYH11-E	CGGGAGGAAATGGAGGCCAAGGCGAACC	D-3abc
			CBFB	CACGCGAATTTGAAGATAGAGACAGGTCTCA	D-4abc
			MYH11-A	ACCCAGATGGAGGAGATGAAGACGCAGC	D-5abc
			MYH11-DE	GACACCCAGGAGTTGCTTCAAGAA	D-6abc

t(12;21)(p13;q22)	*TEL-AML1 *	*TEL-AML1*ex2	TEL-AML1ex2	ATTGGGAGAATAGCAGAATGCATACTTGGAATG	G-1abc
		*TEL-AML1*ex3	TEL-AML1ex3	ATTGGGAGAATAGCAGATGCCAGCACGAGC	G-2abc
			TEL	ATCGGGAAGACCTGGCTTACATGA	G-3abc
			AML1ex3	GCCGCTTCACGCCGCCTTCCACCGC	G-4abc

t(1;19)(q23;p13)	*E2A-PBX1 *	I	E2A-PBX1-I	CCCGACTCCTACAGTGTTTTGAGTATCCGAGG	H-1abc
		Ia	E2A-PBX1-Ia	TACAGTGATGAAAGTGTTCGGTCACCTGGAACCTTTTTG	H-2abc
			E2A	CCTCAGGTTTCACCGGCCCCATGTCACT	H-3abc
			PBX1	TGGACAACATGCTGTTAGCGGAAGGCGTGG	H-4abc

t(9;22)(q34;q11)	*BCR-ABL *	p190	p190	TTCCATGGAGACGCAGAAGCCCTTCAGCGGC	J-1abc
		p210 b2a2	p210-b2a2	TGACCATCAATAAGGAAGAAGCCCTTCAGCG	K-1abc
		p210 b3a2	p210-b3a2	CTGGATTTAAGCAGAGTTCAAAAGCCCTTCAGCGGC	K-2abc
		p230	p230	AGCCTTCGACGTCAAAGCCCTTCAGCG	K-4abc
			BCR190	GGGCGTCCGCAAGACCGGGCAGATCTGG	J-2abc
			BCR210	AGAACATCCGGGAGCAGCAGAAGAAGTGTTT	K-3abc
			BCR230	TGGAGGAGATCGAGCGCCGAGGCATGGAGG	K-5abc
			ABL	CCAAGGCTGGGTCCCAAGCAACTACATCACG	K-6abc

del(1)(p32;p32)	*SIL-TAL1 *	I	SIL-TAL1-I	CGCGGAAGTTGCGGATGACCGAGCGGC	I-1abc
		II	SIL-TAL1-II	CGCGGAAGTTGCGATCGCCCAGGACCA	I-2abc
		III	SIL-TAL1-III	CCTCCCAAAATGCTGATCGCCCAGGACCA	I-3abc
			SIL	GGCTCCCGCTCCTACCCTGCAAACAGA	I-4abc
			TAL1	GCCGAGCGAGGCGGCTCGCAGTGACCC	I-5abc

t(4;11)(q21;q23)	*MLL-AF4 *		MLL	CCCAAAACCACTCCTAGTGAGCCCA	F-1abc
			AF4	TCAAAAACTCACTCAAATTCTCAGCAAG	F-2abc

t(9;11)(p22;q23)	*MLL-AF9 *		AF9	ACCTGGAAACATCTGGAACATCCTGAGGA	E-1abc

t(11;19)(q23;p13.3)	*MLL-ENL *		ENL	GGGTTCACTCACGACTGGATGGTGTTTGTCCG	E-2abc

t(11;19)(q23;p13.1)	*MLL-ELL *		ELL	CCAGCAGTATGTCTCCAGTCATGGGGAAGT	E-3abc

t(6;11)(q27;q23)	*MLL-AF6 *		AF6	TTCGACCTGATATGCGAATGCTGTCCTCTC	E-4abc

t(10;11)(p12;q23)	*MLL-AF10 *	A:2222	AF10-A	GCTTACAGATTCGCTATGATCAACCAGGCA	E-5abc
		B:979	AF10-B	GTTTCAGAGACTAGAGGGTCAGAGGGCA	E-6abc

	*GUS *		GUS	CAGTCACCGACGAGAGTGCTGGGGA	L-1abc, 3abc, 5abc

**Table 4:** Reproducibility of operators and experiments.

Operator	RNA	Experiment 1	Experiment 2	Experiment 3	Reproducibility of experiments	
Operator 1	Kasumi-1	*AML1-ETO *	*AML1-ETO *	*AML1-ETO *		
	THP-1	*MLL-AF9 *	*MLL-AF9 *	*MLL-AF9 *	100%	
	REH	*TEL-AML1*ex2	*TEL-AML1*ex2	*TEL-AML1*ex2		
	HL-60	None	None	None		

Operator 2	Kasumi-1	*AML1-ETO *	*AML1-ETO *	*AML1-ETO *		
	THP-1	*MLL-AF9 *	*MLL-AF9 *	*MLL-AF9 *	100%	100%
	REH	*TEL-AML1*ex2	*TEL-AML1*ex2	*TEL-AML1*ex2		
	HL-60	None	None	None		

Operator 3	Kasumi-1	*AML1-ETO *	*AML1-ETO *	*AML1-ETO *		
	THP-1	*MLL-AF9 *	*MLL-AF9 *	*MLL-AF9 *	100%	
	REH	*TEL-AML1*ex2	*TEL-AML1*ex2	*TEL-AML1*ex2		
	HL-60	None	None	None		

Reproducibility of operators		100%	100%	100%		
			100%			

**Table 5:** Summary of translocations detected by multiplex RT-PCR-microarray method and clinic diagnostic analysis.

Translocations	Multiplex RT-PCR-microarrays			Clinic diagnosis^a^		
	AML	ALL	CML	AML	ALL	CML
t(8;21) *AML1-ETO *	8			8		
t(15;17) *PML-RARA *	10			10		
inv(16) *CBFB-MYH11 *	5			5		
t(9;11) *MLL-AF9 *	1			1		
t(11;19) *MLL-ENL *	1			1		
t(11;19) *MLL-ELL *	1			1		
t(6;11) *MLL-AF6 *	1			1		
t(10;11) *MLL-AF10 *	1			1		
t(4;11) *MLL-AF4 *		4			4	
t(12;21) *TEL-AML1 *		18			18	
t(1;19) *E2A-PBX1 *		3			3	
t(9;22) *BCR-ABL* p190		2			2	
t(9;22) *BCR-ABL* p210			8			8
Other translocations				4	3	
Total number of patients with translocations	28	27	8	32	30	8
Total number of patients	74	115	11	74	115	11

^a^Clinic diagnosis was carried out by either cytogenetic, FISH, or RT-PCR analysis.

## References

[1] Vardiman JW, Harris NL, Brunning RD (2002). The World Health Organization (WHO) classification of the myeloid neoplasms. *Blood*.

[2] Vardiman JW, Thiele J, Arber DA (2009). The 2008 revision of the World Health Organization (WHO) classification of myeloid neoplasms and acute leukemia: rationale and important changes. *Blood*.

[3] Look AT (1997). Oncogenic transcription factors in the human acute leukemias. *Science*.

[4] Gabert J, Beillard E, van der Velden VHJ (2003). Standardization and quality control studies of ’real time’ quantitative reverse transcriptase polymerase chain reaction of fusion gene transcripts for residual disease detection in leukemia—a Europe Against Cancer Program. *Leukemia*.

[5] Raanani P, Ben-Bassat I (2004). Detection of minimal residual disease in acute myelogenous leukemia. *Acta Haematologica*.

[6] Pui C-H, Relling MV, Downing JR (2004). Mechanisms of disease: acute lymphoblastic leukemia. *The New England Journal of Medicine*.

[7] Pallisgaard N, Hokland P, Riishøj DC, Pedersen B, Jørgensen P (1998). Multiplex reverse transcription-polymerase chain reaction for simultaneous screening of 29 translocations and chromosomal aberrations in acute leukemia. *Blood*.

[8] Salto-Tellez M, Shelat SG, Benoit B (2003). Multiplex RT-PCR for the detection of leukemia-associated translocations: validation and application to routine molecular diagnostic practice. *Journal of Molecular Diagnostics*.

[9] King RL, Naghashpour M, Watt CD, Morrissette JJD, Bagg A (2011). A comparative analysis of molecular genetic and conventional cytogenetic detection of diagnostically important translocations in more than 400 cases of acute leukemia, highlighting the frequency of false-negative conventional cytogenetics. *American Journal of Clinical Pathology*.

[10] Wallace J, Zhou Y, Usmani GN (2003). BARCODE-ALL: accelerated and cost-effective genetic risk stratification in acute leukemia using spectrally addressable liquid bead microarrays. *Leukemia*.

[11] Nasedkina T, Domer P, Zharinov V, Hoberg J, Lysov Y, Mirzabekov A (2002). Identification of chromosomal translocations in leukemias by hybridization with oligonucleotide microarrays. *Haematologica*.

[12] Nasedkina TV, Zharinov VS, Isaeva EA (2003). Clinical Screening of Gene Rearrangements in Childhood Leukemia by Using a Multiplex Polymerase Chain Reaction-Microarray Approach. *Clinical Cancer Research*.

[13] Maroc N, Morel A, Beillard E (2004). A diagnostic biochip for the comprehensive analysis of MLL translocations in acute leukemia. *Leukemia*.

[14] Giusiano S, Formisano-Treziny C, Benziane A (2010). Development of a biochip-based assay integrated in a global strategy for identification of fusion transcripts in acute myeloid leukemia: a work flow for acute myeloid leukemia diagnosis. *International Journal of Laboratory Hematology*.

[15] Beillard E, Pallisgaard N, van der Velden VHJ (2003). Evaluation of candidate control genes for diagnosis and residual disease detection in leukemic patients using ’real-time’ quantitative reverse-transcriptase polymerase chain reaction (RQ-PCR)—a Europe against cancer program. *Leukemia*.

[16] Pui C-H, Robison LL, Look AT (2008). Acute lymphoblastic leukaemia. *The Lancet*.

[17] Aricò M, Valsecchi MG, Camitta B (2000). Outcome of treatment in children with Philadelphia chromosome-positive acute lymphoblastic leukemia. *The New England Journal of Medicine*.

[18] Grimwade D, Hills RK (2009). Independent prognostic factors for AML outcome. *Hematology*.

[19] Aspland SE, Bendall HH, Murre C (2001). The role of E2A-PBX1 in leukemogenesis. *Oncogene*.

[20] Pui C-H, Boyett JM, Rivera GK (2000). Long-term results of total therapy studies 11, 12 and 13A for childhood acute lymphoblastic leukemia at St Jude children’s research hospital. *Leukemia*.

[21] Schrappe M, Aricò M, Harbott J (1998). Philadelphia chromosome-positive (Ph+) childhood acute lymphoblastic leukemia: good initial steroid response allows early prediction of a favorable treatment outcome. *Blood*.

[22] Pui C-H, Gaynon PS, Boyett JM (2002). Outcome of treatment in childhood acute lymphoblastic leukaemia with rearrangements of the 11q23 chromosomal region. *The Lancet*.

[23] Nachman JB, Heerema NA, Sather H (2007). Outcome of treatment in children with hypodiploid acute lymphoblastic leukemia. *Blood*.

[24] Rubnitz JE, Raimondi SC, Tong X (2002). Favorable impact of the t(9;11) in childhood acute myeloid leukemia. *Journal of Clinical Oncology*.

[25] Shi RZ, Morrissey JM, Rowley JD (2003). Screening and quantification of multiple chromosome translocations in human leukemia. *Clinical Chemistry*.

