# Supplementary material for: A Pipeline with Multiplex Reverse Transcription Polymerase Chain Reaction and Microarray for Screening of Chromosomal Translocations in Leukemia
Source: Biomed Res Int. 2013 Oct 8;2013:135086. doi: 10.1155/2013/135086 (PMC3816023; doi:10.1155/2013/135086)
Supplement: Supplementary file 1 — Supplementary Text 1: Methods for clinic diagnosis of 200 leukemia samples, including cytogenetic, FISH and RT-PCR analysis. Supplementary Figure 1: Results from the multiplex RT-PCR combined with microarray for positive controls. Supplementary Table 1: Multiplex RT-PCR - microarray and clinic diagnosis of 200 leukemia samples. [file 135086.f1.docx]

**SUPPLEMENTARY TEXT 1**

Methods for clinic diagnosis of 200 leukemia samples

1. Cytogenetic analysis

For each patient a bone marrow sample was collected at diagnosis and mononuclear cells were separated by density-gradient centrifugation with Ficoll solution. Chromosomes were G-banded on unstimulated bone marrow cells after a 24-hour culture. Karyotypes were classified according to International System for Human Cytogenetic Nomenclature (ISCN, 2005). Inclusion in the study required the analysis of ≥20 metaphase cells per patient.

2. Fluorescence *in situ* hybridization (FISH)

FISH was carried out using the direct fluorescence method on fixed cytogenetic specimens of bone marrow samples following protocols supplied by Vysis Inc./Oncor Inc. Signals were observed under epifluorescent microscopy using a dual/triple band pass fluorescent microscope. In every specimen, a minimum of 200 interphase cells and 10-20 metaphase cells were analyzed.

3. RT-PCR

Total RNA was extracted from bone marrow cells using TRIzol reagent (Invitrogen). First-strand cDNA was synthesized from 1 µg total RNA using M-MLV reverse transcriptase (Promega) or Superscript II reverse transcriptase (Invitrogen) with random hexamers according to the manufacturer’s instructions. The fusion transcripts were detected by nested RT-PCR. The results were determined by 2% agarose gel electrophoresis.


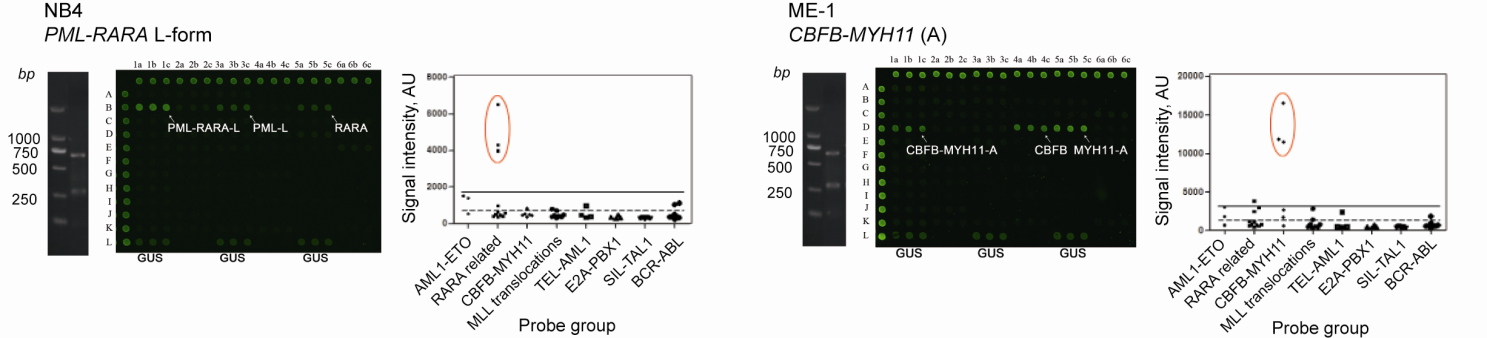

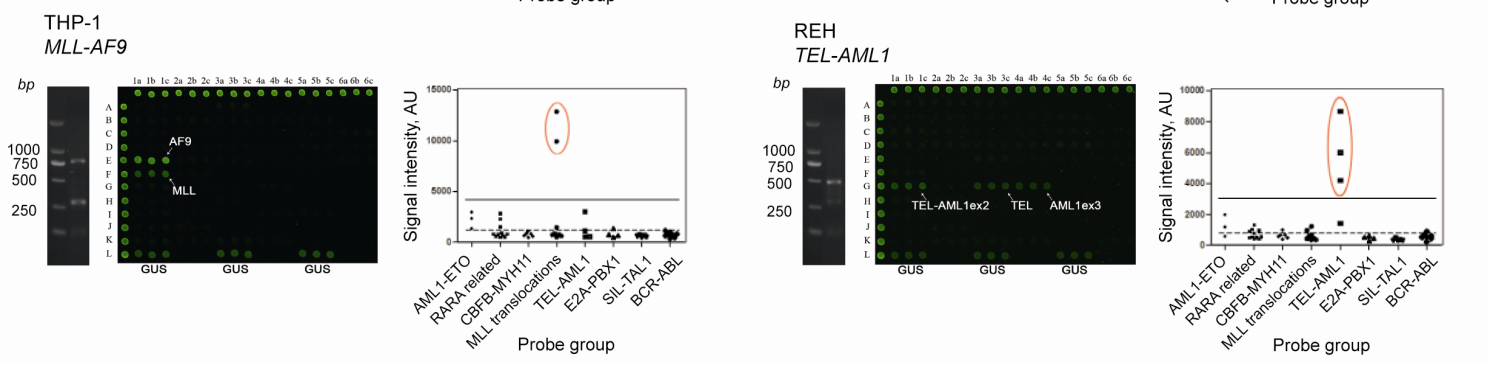

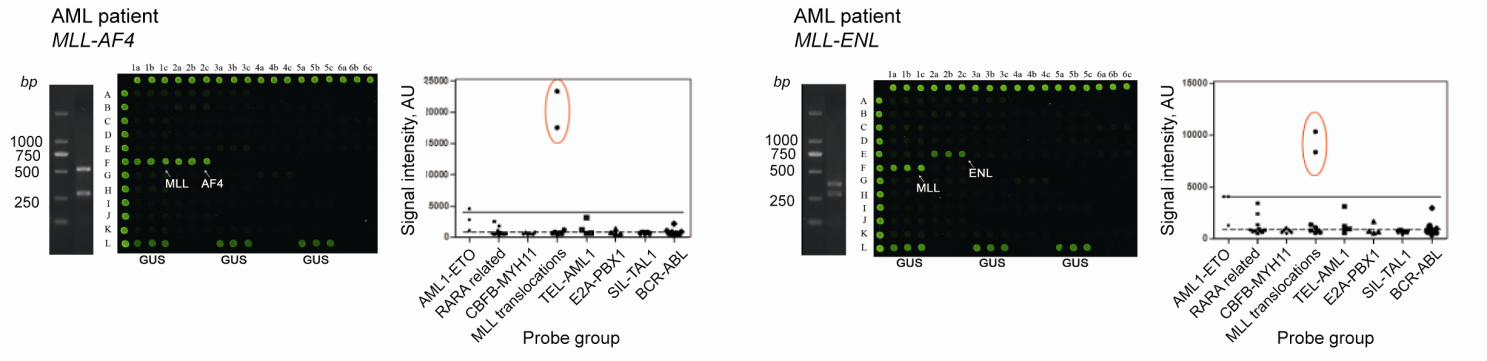

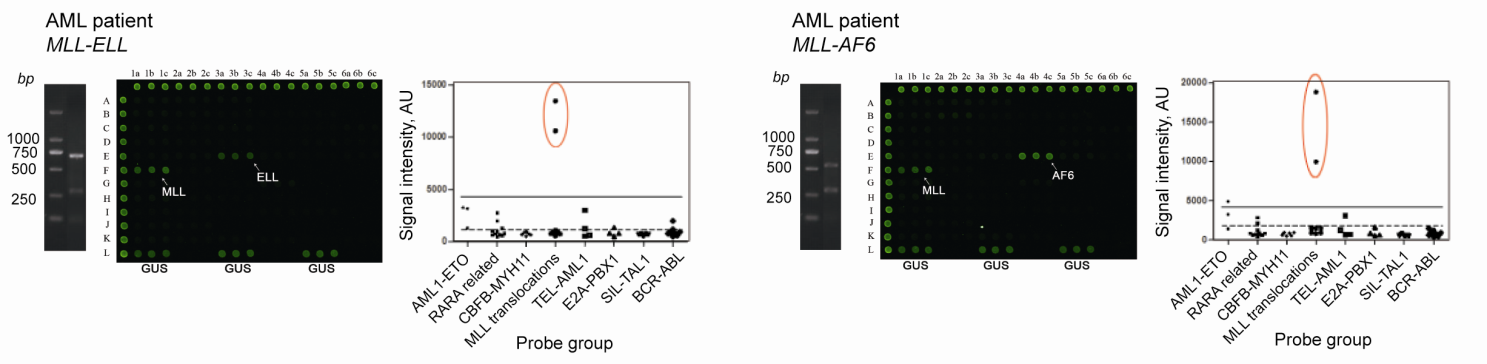

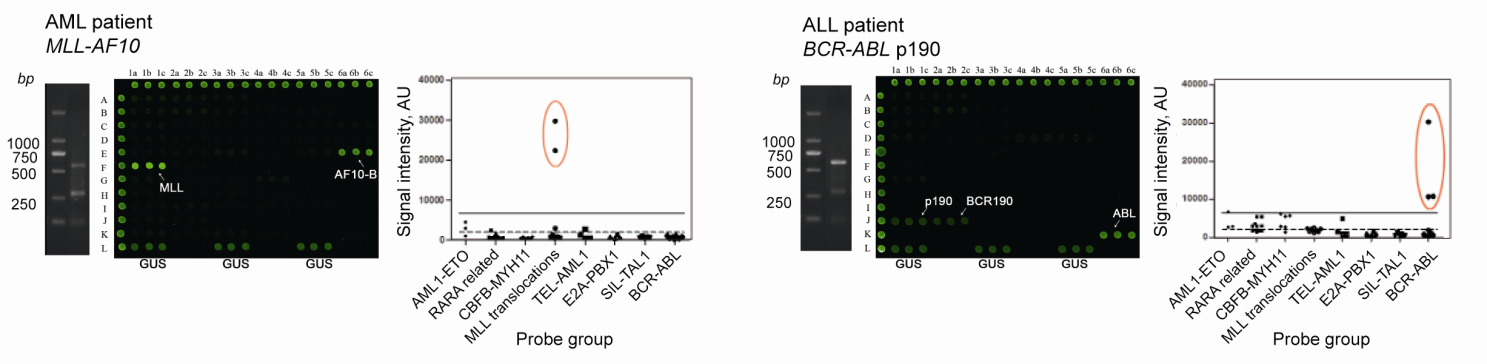

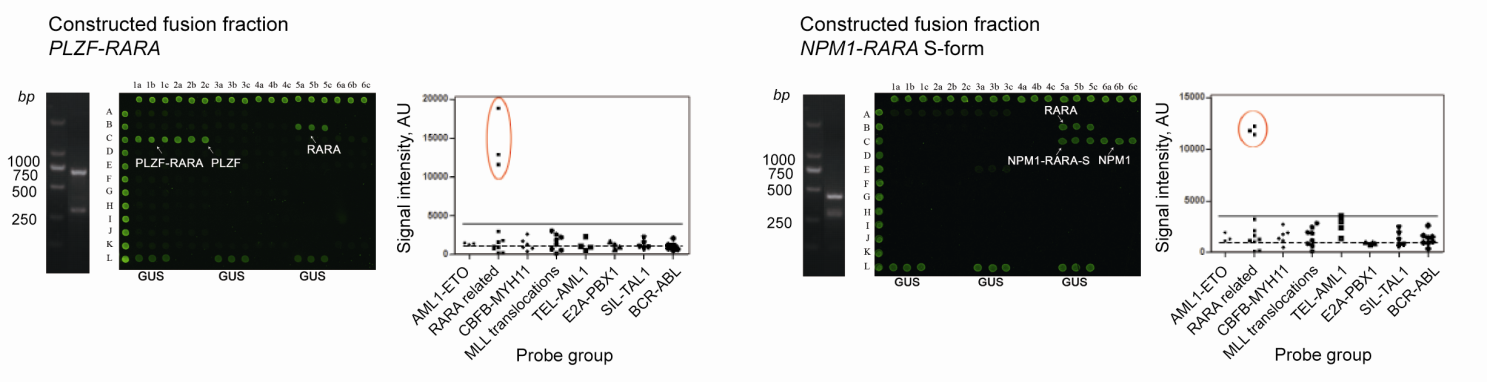

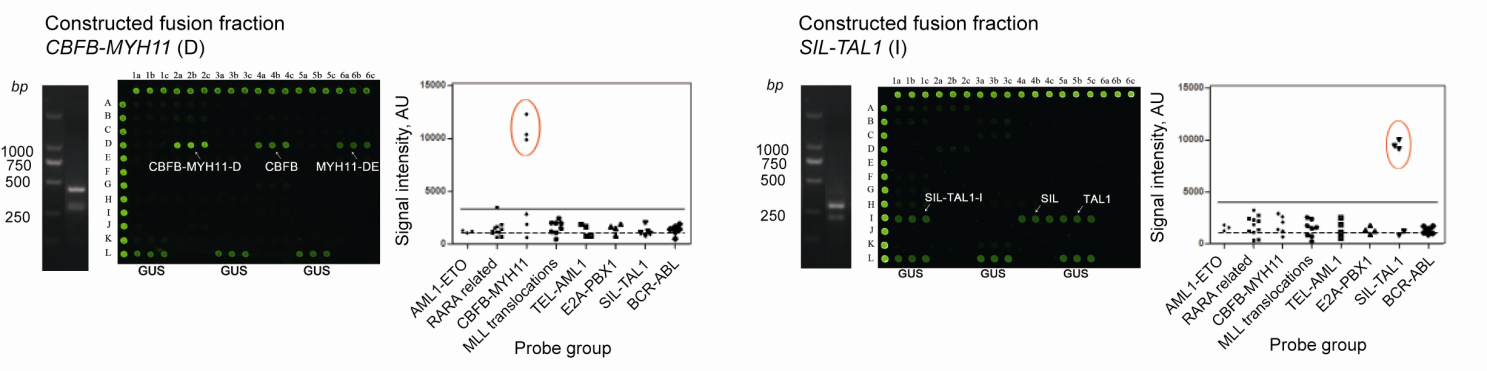

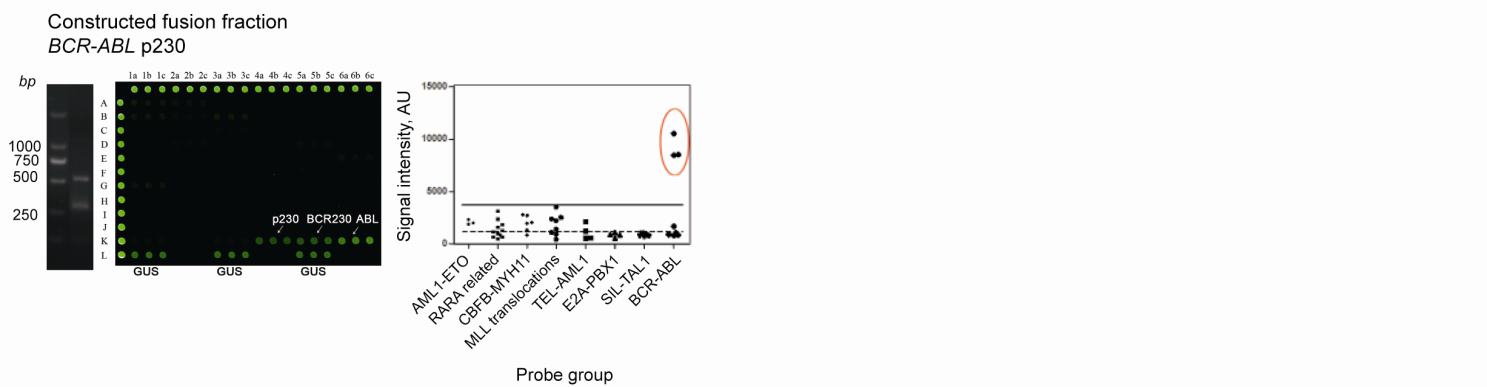


**SUPPLEMENTARY FIGURE 1: Results from the multiplex RT-PCR combined with microarray for positive controls (four leukemic cell lines, six patient samples and five constructed fusion fractions).** HL-60 and H_2_O were used as negative controls.

**SUPPLEMENTARY TABLE 1: Multiplex RT-PCR - microarray and clinic diagnosis of 200 leukemia samples**

| Patient | Leukemia Subtype | Multiplex RT-PCR - Microarray | Clinic Diagnosis^a^ |
| --- | --- | --- | --- |
| 1 | AML | ND | ND |
| 2 | AML | *AML1-ETO* | *AML1-ETO* |
| 3 | AML | ND | ND |
| 4 | AML | *PML-RARA* L-form | *PML-RARA* L-form |
| 5 | AML | ND | ND |
| 6 | AML | ND | ND |
| 7 | AML | *CBFB-MYH11*(A) | *CBFB-MYH11*(A) |
| 8 | AML | *AML1-ETO* | *AML1-ETO* |
| 9 | AML | ND | dup*MLL*(11q23) |
| 10 | AML | ND | ND |
| 11 | AML | ND | ND |
| 12 | AML | *PML-RARA* S-form | *PML-RARA* S-form |
| 13 | AML | ND | ND |
| 14 | AML | ND | ND |
| 15 | AML | *AML1-ETO* | *AML1-ETO* |
| 16 | AML | ND | ND |
| 17 | AML | ND | ND |
| 18 | AML | *CBFB-MYH11*(A) | *CBFB-MYH11*(A) |
| 19 | AML | ND | ND |
| 20 | AML | *CBFB-MYH11*(A) | *CBFB-MYH11*(A) |
| 21 | AML | *PML-RARA* L-form | *PML-RARA* L-form |
| 22 | AML | ND | ND |
| 23 | AML | ND | ND |
| 24 | AML | ND | ND |
| 25 | AML | *PML-RARA* L-form | *PML-RARA* L-form |
| 26 | AML | ND | ND |
| 27 | AML | ND | ND |
| 28 | AML | ND | dup*MLL*(11q23) |
| 29 | AML | ND | ND |
| 30 | AML | ND | ND |
| 31 | AML | *MLL-AF9* | *MLL-AF9* |
| 32 | AML | ND | ND |
| 33 | AML | ND | ND |
| 34 | AML | ND | ND |
| 35 | AML | *AML1-ETO* | *AML1-ETO* |
| 36 | AML | *AML1-ETO* | *AML1-ETO* |
| 37 | AML | ND | ND |
| 38 | AML | *AML1-ETO* | *AML1-ETO* |
| 39 | AML | ND | ND |
| 40 | AML | *PML-RARA* L-form | *PML-RARA* L-form |
| 41 | AML | ND | dup*MLL*(11q23) |
| 42 | AML | ND | ND |
| 43 | AML | ND | ND |
| 44 | AML | ND | ND |
| 45 | AML | *PML-RARA* S-form | *PML-RARA* S-form |
| 46 | AML | ND | ND |
| 47 | AML | ND | ND |
| 48 | AML | *CBFB-MYH11*(A) | *CBFB-MYH11*(A) |
| 49 | AML | ND | dup*MLL*(11q23) |
| 50 | AML | ND | ND |
| 51 | AML | ND | ND |
| 52 | AML | *MLL-ENL* | *MLL-ENL* |
| 53 | AML | ND | ND |
| 54 | AML | *AML1-ETO* | *AML1-ETO* |
| 55 | AML | *AML1-ETO* | *AML1-ETO* |
| 56 | AML | ND | ND |
| 57 | AML | △*PML-RARA* V-form | *PML-RARA* |
| 58 | AML | *PML-RARA* L-form | *PML-RARA* L-form |
| 59 | AML | Neg | ND |
| 60 | AML | ND | ND |
| 61 | AML | *CBFB-MYH11*(A) | *CBFB-MYH11*(A) |
| 62 | AML | ND | ND |
| 63 | AML | *MLL-ELL* | *MLL-ELL* |
| 64 | AML | *PML-RARA* S-form | *PML-RARA* S-form |
| 65 | AML | ND | ND |
| 66 | AML | Neg | ND |
| 67 | AML | *PML-RARA* S-form | *PML-RARA* S-form |
| 68 | AML | Neg | ND |
| 69 | AML | *MLL-AF6* | *MLL-AF6* |
| 70 | AML | ND | ND |
| 71 | AML | ND | ND |
| 72 | AML | *MLL-AF10*(B:979) | *MLL-AF10* |
| 73 | AML | Neg | ND |
| 74 | AML | ND | ND |
|  |  |  |  |
| 101 | ALL | *TEL-AML1*ex2 | *TEL-AML1* |
| 102 | ALL | ND | ND |
| 103 | ALL | *TEL-AML1*ex2 | *TEL-AML1* |
| 104 | ALL | *TEL-AML1*ex2 | *TEL-AML1* |
| 105 | ALL | ND | ND |
| 106 | ALL | ND | ND |
| 107 | ALL | ND | ND |
| 108 | ALL | *TEL-AML1*ex2 | *TEL-AML1* |
| 109 | ALL | ND | ND |
| 110 | ALL | ND | ND |
| 111 | ALL | Neg | ND |
| 112 | ALL | ND | ND |
| 113 | ALL | ND | ND |
| 114 | ALL | ND | ND |
| 115 | ALL | *MLL-AF4* | *MLL-AF4* |
| 116 | ALL | ND | ND |
| 117 | ALL | *BCR-ABL* (e1-a2) | *BCR-ABL* p190 |
| 118 | ALL | ND | ND |
| 119 | ALL | *TEL-AML1*ex2 | *TEL-AML1* |
| 120 | ALL | *TEL-AML1*ex2 | *TEL-AML1* |
| 121 | ALL | ND | ND |
| 122 | ALL | ND | dup*MLL*(11q23) |
| 123 | ALL | ND | ND |
| 124 | ALL | ND | ND |
| 125 | ALL | ND | ND |
| 126 | ALL | *E2A-PBX1* (I) | *E2A-PBX1* |
| 127 | ALL | ND | ND |
| 128 | ALL | *MLL-AF4* | *MLL-AF4* |
| 129 | ALL | ND | ND |
| 130 | ALL | ND | ND |
| 131 | ALL | ND | ND |
| 132 | ALL | ND | ND |
| 133 | ALL | ND | ND |
| 134 | ALL | ND | ND |
| 135 | ALL | ND | ND |
| 136 | ALL | *TEL-AML1*ex2 | *TEL-AML1* |
| 137 | ALL | ND | ND |
| 138 | ALL | Neg | ND |
| 139 | ALL | ND | ND |
| 140 | ALL | ND | ND |
| 141 | ALL | *TEL-AML1*ex2+ *TEL-AML1*ex3 | *TEL-AML1* |
| 142 | ALL | ND | ND |
| 143 | ALL | *E2A-PBX1* (I) | *E2A-PBX1* |
| 144 | ALL | ND | ND |
| 145 | ALL | ND | *DEK-CAN* |
| 146 | ALL | ND | ND |
| 147 | ALL | ND | ND |
| 148 | ALL | *BCR-ABL* (e1-a2) | *BCR-ABL* p190 |
| 149 | ALL | ND | ND |
| 150 | ALL | ND | ND |
| 151 | ALL | Neg | ND |
| 152 | ALL | ND | ND |
| 153 | ALL | ND | ND |
| 154 | ALL | *TEL-AML1*ex2 | *TEL-AML1* |
| 155 | ALL | ND | ND |
| 156 | ALL | ND | ND |
| 157 | ALL | ND | ND |
| 158 | ALL | ND | ND |
| 159 | ALL | *TEL-AML1*ex2 | *TEL-AML1* |
| 160 | ALL | *TEL-AML1*ex2 | *TEL-AML1* |
| 161 | ALL | ND | ND |
| 162 | ALL | ND | ND |
| 163 | ALL | Neg | ND |
| 164 | ALL | ND | ND |
| 165 | ALL | ND | ND |
| 166 | ALL | ND | ND |
| 167 | ALL | Neg | ND |
| 168 | ALL | ND | ND |
| 169 | ALL | *TEL-AML1*ex3 | *TEL-AML1* |
| 170 | ALL | ND | ND |
| 171 | ALL | ND | ND |
| 172 | ALL | ND | ND |
| 173 | ALL | ND | ND |
| 174 | ALL | Neg | ND |
| 175 | ALL | ND | ND |
| 176 | ALL | ND | ND |
| 177 | ALL | ND | ND |
| 178 | ALL | *E2A-PBX1* (Ia) | *E2A-PBX1* |
| 179 | ALL | ND | ND |
| 180 | ALL | ND | ND |
| 181 | ALL | ND | ND |
| 182 | ALL | ND | ND |
| 183 | ALL | Neg | ND |
| 184 | ALL | ND | ND |
| 185 | ALL | ND | ND |
| 186 | ALL | *TEL-AML1*ex2 | *TEL-AML1* |
| 187 | ALL | ND | ND |
| 188 | ALL | ND | ND |
| 189 | ALL | *TEL-AML1*ex2 | *TEL-AML1* |
| 190 | ALL | Neg | ND |
| 191 | ALL | ND | ND |
| 192 | ALL | ND | ND |
| 193 | ALL | ND | ND |
| 194 | ALL | ND | ND |
| 195 | ALL | *TEL-AML1*ex2 | *TEL-AML1* |
| 196 | ALL | ND | ND |
| 197 | ALL | ND | ND |
| 198 | ALL | ND | ND |
| 199 | ALL | *TEL-AML1*ex2+ *TEL-AML1*ex3 | *TEL-AML1* |
| 200 | ALL | ND | ND |
| 201 | ALL | ND | ND |
| 202 | ALL | ND | ND |
| 203 | ALL | *TEL-AML1*ex2 | *TEL-AML1* |
| 204 | ALL | ND | ND |
| 205 | ALL | *MLL-AF4* | *MLL-AF4* |
| 206 | ALL | ND | ND |
| 207 | ALL | ND | dup*MLL*(11q23) |
| 208 | ALL | ND | ND |
| 209 | ALL | ND | ND |
| 210 | ALL | *TEL-AML1*ex2 | *TEL-AML1* |
| 211 | ALL | ND | ND |
| 212 | ALL | ND | ND |
| 213 | ALL | *MLL-AF4* | *MLL-AF4* |
| 214 | ALL | ND | ND |
| 215 | ALL | Neg | ND |
|  |  |  |  |
| 301 | CML | *BCR-ABL* (b3a2) | *BCR-ABL* p210 |
| 302 | CML | *BCR-ABL* (b3a2) | *BCR-ABL* p210 |
| 303 | CML | *BCR-ABL* (b2a2) | *BCR-ABL* p210 |
| 304 | CML | ND | ND |
| 305 | CML | *BCR-ABL* (b3a2) | *BCR-ABL* p210 |
| 306 | CML | ND | ND |
| 307 | CML | *BCR-ABL* (b2a2) | *BCR-ABL* p210 |
| 308 | CML | ND | ND |
| 309 | CML | *BCR-ABL* (b3a2) | *BCR-ABL* p210 |
| 310 | CML | *BCR-ABL* (b2a2) | *BCR-ABL* p210 |
| 311 | CML | *BCR-ABL* (b3a2) | *BCR-ABL* p210 |

^a^ Clinic diagnosis was carried out by either cytogenetic, FISH, or RT-PCR analysis.

Abbreviations: ND, no detection of translocations; Neg, negative
